# Investigation of Prophylactic and Therapeutic Effects of Boric Acid in an Experimental Mechanical Biliary Obstructive Cholestasis Rat Model

**DOI:** 10.1007/s12011-025-04665-3

**Published:** 2025-05-21

**Authors:** Cengiz Kocak, Fatma Emel Kocak, Fatih Kar, Raziye Akcılar, Sezgin Zeren, Ali Cihat Yıldırım, Mehmet Fatih Ekici, Ezgi Kar

**Affiliations:** 1 Department of Pathology, Faculty of Medicine, Kutahya Health Sciences University, Kutahya, Turkey; 2 Department of Medical Biochemistry, Faculty of Medicine, Kutahya Health Sciences University, Kutahya, Turkey; 3 Department of Physiology, Faculty of Medicine, Kutahya Health Sciences University, Kutahya, Turkey; 4 Department of General Surgery, Faculty of Medicine, Kutahya Health Sciences University, Kutahya, Turkey; 5 Department of Nutrition and Dietetics, Faculty of Health Sciences, Kutahya Health Sciences University, Kutahya, Turkey

**Keywords:** Cholestasis, Jaundice, Obstructive, Boric acids

## Abstract

Obstructive jaundice is a serious condition that increases the risk of death from multiple organ failure and sepsis. The aim of this study was to investigate the therapeutic (T) and prophylactic (P) effects of boric acid (BA) in an experimental mechanical biliary obstructive (BO) cholestasis rat model. For this purpose, thirty-two adult male Wistar Albino rats were divided into four groups of eight animals in each group as control, BO, BO + BAP, and BO + BAT. BA was administered intraperitoneally at 100 mg/kg/day before and after bile duct ligation. In order to examine the anti-inflammatory and antioxidant effects of BA as well as the oxidative stress and inflammation caused by BO, serum interleukin-1β (IL-1β), tumor necrosis factor-α (TNF-α), total oxidant and antioxidant status (TOS, TAS), and cytochrome C levels were measured. Serum total bilirubin (T. Bil), direct bilirubin (D. Bil) levels and amylase, gamma glutamyl transferase (GGT), alkaline phosphatase (ALP), aspartate aminotransferase (AST), alanine aminotransferase (ALT) activities were measured to investigate liver functions. Furthermore, the mRNA expression levels of the TNF-α, interleukin-6 (IL-6), caspase-3, and B-cell lymphoma 2 (Bcl-2) genes in liver tissue were examined to investigate the anti-inflammatory and antiapoptotic effects of BA as well as the inflammation and apoptosis caused by BO. Hematoxylin–eosin and Masson’s trichrome staining were performed for histopathological examinations. BO-induced liver damage, oxidative stress, inflammation, and apoptosis were reduced by BA administration. The anti-apoptotic, anti-inflammatory, and antioxidant effects of prophylactic BA application were found to be more effective than therapeutic BA application.

## Introduction

Obstructive jaundice is a critical condition that carries a risk of mortality due to sepsis and multiple organ failure [1]. The main factors contributing to the development of cholestasis are the disruption of bile flow and the resulting hepatic retention of bile acids. Bile acids or their conjugated bile salts accumulate in the liver and systemic circulation as a result of cholestasis [2]. Intrahepatic cholestasis results from impaired bile production by liver cells, whereas obstructive or extrahepatic cholestasis is caused by obstruction of the bile ducts that carry bile from the liver to the intestines. The typical cause of obstructive cholestasis is physical obstruction of the biliary system at the level of the extrahepatic bile ducts, usually brought on by a tumor or stone [3]. Due to the strong detergent properties of hydrophobic bile acids, bile acid accumulation in the liver causes oxidative stress, mitochondrial damage, inflammation, activation of hepatic stellate cells, and hepatocyte death [4]. Proinflammatory cytokines are released, and thus an inflammatory reaction is initiated, immune cells such as neutrophils are recruited and damage hepatic parenchymal cells, resulting in hepatic dysfunction, fibrosis, cirrhosis, and liver failure [5].

The unmet demand for efficient agents for liver diseases continues to be a health barrier despite significant advancements in modern medicine. Investigating alternative drugs is essential for the prevention and/or treatment of liver disease. Although boron has been a chemical element of interest since ancient times, only a few compounds containing boron were used for medical purposes before the twenty-first century. Among these, boric acid (BA) has been investigated in various therapeutic applications. Nowadays, there is an increase in research on new boron-containing compounds as a potential agent in the prevention and treatment of various diseases [6]. The nutritional role of BA, identified as a weak monobasic Lewis acid of boron, in human and animal metabolism became clear in the 1980s [7]. The half-life of BA, which is distributed into body fluids after being absorbed from the gastrointestinal tract, is approximately 24 h. BA accumulates at low concentrations in some organs, such as the brain, bone, and liver until it is excreted through the kidneys [8]. Various experimental and clinical studies have demonstrated the anti-inflammatory, anti-apoptotic, and antioxidant properties of BA [8–13]. Additionally, it has been proposed that BA increases the body’s glutathione deposits and prevents oxidative damage by blocking other reactive oxygen species [14].

Based on the aforementioned information, the purpose of this study was to examine the therapeutic and prophylactic effects of BA in a rat model of experimental mechanical biliary obstructive cholestasis. The effects of BA on biliary obstructive cholestasis were investigated by measuring some biochemical, oxidative, apoptotic, and inflammatory parameters. Morphological changes were also examined histopathologically.

## Materials and Methods

### Ethics Statement

After being approved by the Animal Experiments Local Ethics Committee, this experimental investigation was conducted at the Experimental Animal Center of Kutahya Health Sciences University in Kutahya, Turkey (Decision Number: E-12650661–604.01.02–64496). Rats were acquired from the Experimental Animal Center at Kutahya Health Sciences University. Every experiment was conducted in compliance with the Institute of Laboratory Animal Resources Commission on Life Sciences National Research Council’s Guide for the Care and Use of Laboratory Animals [15].

### The Preparation of Chemical Materials

BA (MW: 61.83, purity 99.5, Lot Number: 57H0655) was purchased from Sigma-Aldrich^®^ Lab & Production Materials (Sigma-Aldrich Co. LLC., St. Louis, MO). The compound was dissolved in physiological saline solution and freshly prepared before administration. The BA solution was administered intraperitoneally (i.p.) at a dose of 100 mg/kg/day in 0.5-mL physiological saline before and after bile duct ligation. The dose of BA was preferred according to previous studies [16].

### Animals

Thirty-two adult male Wistar Albino rats (Kutahya Health Sciences University Experimental Animal Laboratory, Kütahya, Turkey) weighing 250–300 g were used. All rats were housed in transparent polycarbonate cages with a 12-:12-h light–dark cycle at 21 °C and provided ad libitum access to fresh water and standard rat chow. All rats were fed commercial standard pellet chow composed of 89.91% dry matter containing the following ingredients: 6.3% crude fiber, 24% crude protein, 4.9% crude fat, 6.1% crude ash, 28.7% starch, 4.7% sugar, 11.3% moisture, 1–2% vitamins and minerals, 3% trace elements, and 53.4% nitrogen-free extractives (metabolic energy 2700 kcal/kg). Five days previous to the behavioral investigation, the animals were acclimated to the laboratory environment, and they remained there until the study was finished.

### Experimental Study Design

Thirty-two male rats (n = 8 for each group) were randomly divided into four experimental groups and they were organized as follows:

- Group 1, Control (C): Only laparotomy was performed. No treatment was applied to the rats in this group.
- Group 2, Biliary Obstruction (BO): Group 2 was administered 0.5-mL normal saline i.p. once a day before and after bile duct ligation.
- Group 3, BO + BA prophylaxis (BAP): Group 3 was administered BA 100 mg/kg i.p. once daily before bile duct ligation.
- Group 4, BO + BA treatment (BAT): Group 4 was administered BA 100 mg/kg i.p. once daily after bile duct ligation.

### Experimental Mechanical Biliary Obstructive Cholestasis Model

The i.p. injection of 10 mg/kg xylazine hydrochloride (Rompun, Bayer, Istanbul, Turkey) and 90 mg/kg ketamine (Ketalar, Pfizer, Istanbul, Turkey) was used to anesthetize the rats after they had been weighed. Once an acceptable level of anesthesia was achieved, the rats were placed on a homeothermic table to maintain a steady body temperature of 37 ± 1 °C. A povidone-iodine solution was used to sterilize the anterior abdominal wall after shaving. Following a 1–2 cm midline laparotomy, the common bile duct was located, separated from the adjacent portal vein and hepatic artery and doubly ligated with 4/0 silk sutures. The peritoneal cavity was rinsed with 0.9% NaCl solution, and the abdominal organs were replaced in their physiological positions. Following the surgeries, 4/0 silk sutures were used to close abdominal wounds in two layers. The animals were cared for and fed on a regular basis for seven days as mentioned earlier. On the eighth postoperative day, under general anesthesia, the initial incision was used to perform the re-laparotomy, and the rats were sacrificed [17].

### Tissue Preparation and Blood Sampling

Following anesthesia with Ketamine/Xylazine HCl (90 mg/kg/10 mg/kg, i.p.) at the conclusion of the experiment, the animals in each group were sacrificed. Cardiac puncture blood samples were transferred to suitable separator gel tubes for biochemical examinations. For 15 min, blood samples were centrifuged at 3500 rpm. Then, until biochemical testing, serum samples were kept at −80 °C. After obtaining liver tissue samples, any red blood cells or clots were washed away with cold heparinized phosphate-buffered saline. For molecular biological analyses, tissue samples were placed in liquid nitrogen and kept at −80 °C until analysis. For histopathologic examinations, some of the liver tissue samples were preserved in 10% buffered formalin.

## Biochemical Analyses

### Measurement of Serum Total Bilirubin (T. Bil), Direct Bilirubin (D. Bil) Levels and Amylase, Gamma Glutamyl Transferase (GGT), Alkaline Phosphatase (ALP), Aspartate Aminotransferase (AST), Alanine Aminotransferase (ALT) Activities

Serum T. Bil, D. Bil levels and AST, ALT, ALP, GGT, amylase activities were measured on the Beckman Coulter AU680 analyzer (Beckman Coulter, Miami, FL, USA) using commercial reagents (Beckman Coulter, Miami, FL, USA).

### Measurement of Serum Total Antioxidant Status (TAS) and Total Oxidant Status (TOS) Levels

The Beckman Coulter AU680 analyzer (Beckman Coulter, Miami, FL, USA) was used to quantify serum TAS and TOS levels using commercial reagents (Rel Assay Diagnostic, Gaziantep, Turkey) based on innovative automated measuring techniques created by Erel [18, 19].The unit of measurement for TAS levels was mmol Trolox Eq/L. The unit of measurement for TOS levels was μmol H_2_O_2_ Eq/L.

### Calculation of The Oxidative Stress Index (OSI)

The OSI, a measure of the level of oxidative stress, was established as the percentage ratio of TOS to TAS. The following formula was used to do the computation after converting the TAS unit, mmol Trolox equivalent/L, to μmol Trolox equivalent/L: OSI = [(TOS, μmol H_2_O_2_ Eq/L)/(TAS, μmol Trolox Eq/L) × 100] [20].

### Measurement of Serum Interleukin 1 beta (IL-1β), Tumor Necrosis Factor Alpha (TNF-α), and Cytochrome C Levels

Commercial enzyme-linked immunosorbent assay (ELISA) kits (Sunred Biological Technology Co., Ltd., Shanghai, PRC eBioscience, Bender MedSystems GmbH, Vienna, Austria) and a microplate reader (BMG Labtech Spectrostar Nano, GmbH, Ortenberg, Germany) were used to measure the levels of serum cytochrome C, TNF-α, and IL-1β.

### Real-time Polymerase Chain Reaction (RT-PCR) Analysis

Following the manufacturer’s instructions, total RNA was extracted from liver tissues using the GeneJET RNA Purification Kit (Thermo, Cat. No: # K0732). Using the EasyScript™ cDNA Synthesis Kit (abm), 4 mg of total RNA was reverse-transcribed into complementary DNA (cDNA) in accordance with the directions. Then, quantitative RT-PCR was done using the specific primer pairs listed in Table 1 and EvaGreen 2X qPCR Master Mix to detect signals. The RT-PCR was performed using the Applied Biosystems™ StepOnePlus™ Real-Time PCR System (Thermo Fisher Scientific, Leicestershire, England) with the following conditions: 95 °C for 15 min, followed by 40 cycles at 95 °C for 30 s, 60 °C for 30 s and 72 °C for 30 s. The amount of mRNA for each gene was normalized by β-actin, and the relative expression levels were calculated by using the 2-^ΔΔCt^ method.

**Table 1:** Primers used for real-time PCR analysis

Gene	Sense strand sequence	Anti-sense strand sequence
TNF-α	CCA CCA CGC TCT TCT GTC TAC	GCT ACG GGC TTG TCA CTC G
IL-6	CTT CCA GCC AGT TGC CTT CTT G	TGG TCT GTT GTG GGT GGT ATC C
Caspase 3	GAC TGC GGT ATT GAG ACA GA	CGA GTG AGG ATG TGC ATG AA
Bcl-2	AGA TGA AGA CTC CGC GCC	GTA GTG AGA CCC ACG TAT GGA CC
β-actin	CTA TCG GCA ATG AGC GGT TCC	TGT GTT GGC ATA GAG GTC TTT ACG

*TNF*-α; tumor necrosis factor-alpha, *IL-6*; Interleukin-6, *Bcl-2*; B-cell lymphoma 2

### Histopathologic Examinations

Samples of liver tissue were preserved in 10% formalin, embedded in paraffin, cut into 4-mm sections, put on slides, and stained with hematoxylin and eosin (H&E) and Masson’s trichrome. Thereafter, the slides were seen under a light microscope (Olympus BX51, Tokyo, Japan) by a pathologist who was not aware of the treatment groups. Slides were evaluated according to the modified hepatitis activity index of Ishak et al. [21]. Ductal proliferation was scored ranging from 0 to 3; no ductal proliferation (grade 0), limited moderate ductal proliferation in the portal region (grade 1), significant ductular proliferation in the bridges connecting the portals (grade 2). The histopathological fibrosis was scored ranging from 0 to 4; no fibrosis (grade 0), portal fibrosis (grade 1), septal fibrosis (grade 2), incomplete cirrhosis (grade 3), and complete cirrhosis (grade 4).

### Statistical Analysis

The data were analyzed by SPSS version 16.0 (SPSS Co., Chicago, IL, USA) for Windows. Sample size was calculated with G-Power. The Shapiro–Wilk test was used to determine whether the numerical data were normally distributed. Since the whole data were distributed normally, the data were expressed as mean ± standard deviation (SD). The ANOVA test was used to compare numerical variables among groups. To choose the post-hoc test, the Levene statistic was used to conduct a homogeneity test of variances of variables. The Bonferroni post-hoc test was used to evaluate the multiple comparisons of variables since whole variables passed the homogeneity of variances test. The threshold for statistical significance was set at p-value < 0.05.

## Results

### Serum T. Bil, D. Bil Levels and Amylase, GGT, ALP, AST, ALT Activities

Differences in serum amylase, GGT, ALP, AST, ALT activities and serum T. Bil, D. Bil levels between assay groups are represented in Table 2. Significant differences were observed among groups for T. Bil and D. Bil levels (*p* < *0.0001, p* < *0.0001*, respectively). The BO procedure caused significant increases in T. Bil and D. Bil levels (*p* < *0.05*) in comparison with the control group. BA decreased T. Bil and D. Bil levels compared to the BO group (*p* < *0.05*). The BO procedure caused significant increases in serum amylase, GGT, ALP, AST, and ALT activities. BA decreased serum amylase, GGT, ALP, AST, and ALT activities.

**Table 2:** Comparisons of hepatobiliary injury markers between assay groups

Parameters (mean ± SD)	Group 1 (*n* = 8)	Group 2 (*n* = 8)	Group 3 (*n* = 8)	Group 4 (*n* = 8)	*p*
T. Bilirubin (mg/dL)	0.09 ± 0.03	9.30 ± 3.77^a^	1.11 ± 1.22^b^	1.87 ± 2.12^b^	< 0.0001
D. Bilirubin (mg/dL)	0.02 ± 0.01	6.18 ± 2.01^a^	0.99 ± 0.56^b^	1.14 ± 0.51^b^	< 0.0001
AST (U/L)	87 ± 39	346 ± 73^a^	194 ± 98^ab^	203 ± 77^ab^	< 0.0001
ALT (U/L)	77 ± 21	320 ± 76^a^	157 ± 32^ab^	182 ± 54^ab^	< 0.0001
GGT (U/L)	125 ± 46	413 ± 91^a^	226 ± 73^ab^	237 ± 74^ab^	< 0.0001
ALP (U/L)	81 ± 14	424 ± 108^a^	202 ± 96^ab^	209 ± 86^ab^	< 0.0001
Amylase (U/L)	32 ± 8	440 ± 154^a^	148 ± 32^ab^	173 ± 55^ab^	< 0.0001

*GGT*; Gamma glutamyl transferase, *ALP*; Alkaline phosphatase, *AST*; Aspartate aminotransferase, *ALT*; Alanine aminotransferase

Group 1: Control, Group 2: Biliary Obstruction (BO), Group 3: BO + Boric acid prophylaxis, Group 4: BO + Boric acid treatment. Results are expressed as mean ± standard deviation (SD). Since all data were normally distributed, the comparisons of variables among groups were analyzed with the oneway-ANOVA; The P-value shows the differences among all groups. Since all data were passed the homogeneity of variances test, the multiple comparisons between two groups were analyzed with the Bonferroni-post-hoc test; ^a^ = *P* < 0.05 compared with the control group (group 1), ^b^ = *P* < 0.05 compared with the BO group (group 2), ^c^ = *P* < 0.05 compared with the BO + Boric acid prophylaxis group (group 3)

### Serum TOS, TAS, and OSI Levels

Differences in serum TAS, TOS, and OSI levels between assay groups are represented in Table 3. Significant differences were observed among groups for TAS, TOS, and OSI values (*p* < *0.0001, p* < *0.0001, p* < *0.0001*, respectively). The BO procedure caused significant increases in TOS and OSI levels (*p* < *0.05*) and significant decreases in TAS levels (*p* < *0.05*) in comparison with those caused by the control group. Although preoperative and postoperative BA application was observed to decrease TOS and OSI levels and increase TAS levels compared to the BO group (*p* < *0.05*), prophylactic BA application was observed to be more effective.

**Table 3:** Comparisons of oxidative stress parameters between assay groups

Parameters (mean ± SD)	Group 1 (*n* = 8)	Group 2 (*n* = 8)	Group 3 (*n* = 8)	Group 4 (*n* = 8)	*p*
TAS (mmol Trolox Eq/L)	0.97 ± 0.02	0.54 ± 0.008^a^	0.79 ± 0.01^ab^	0.58 ± 0.01^abc^	< 0.0001
TOS (μmol H_2_O_2_ Eq/L)	5.57 ± 0.11	11.27 ± 0.23^a^	8.62 ± 0.08^ab^	9.42 ± 0.09^abc^	< 0.0001
OSI (Arbitrary units)	5.76 ± 0.23	20.86 ± 0.61^a^	10.98 ± 0.26^ab^	16.40 ± 0.52^abc^	< 0.0001

*TAS*; Total antioxidant status, *TOS*; Total oxidant status, *OSI*; Oxidative stress index

Group 1: Control, Group 2: Biliary Obstruction (BO), Group 3: BO + Boric acid prophylaxis, Group 4: BO + Boric acid treatment. Results are expressed as mean ± standard deviation (SD). Since all data were normally distributed, the comparisons of variables among groups were analyzed with the oneway-ANOVA; The P-value shows the differences among all groups. Since all data were passed the homogeneity of variances test, the multiple comparisons between two groups were analyzed with the Bonferroni-post-hoc test; ^a^ = *P* < 0.05 compared with the control group (group 1), ^b^ = *P* < 0.05 compared with the BO group (group 2), ^c^ = *P* < 0.05 compared with the BO + Boric acid prophylaxis group (group 3)

### Serum IL-1β, TNF-α, and Cytochrome C Levels

Differences in serum IL-1β, TNF-α, and cytochrome C levels between assay groups are represented in Table 4. Significant differences were observed among groups for IL-1β, TNF-α, and cytochrome C (*p* < *0.0001, p* < *0.0001*, *p* = *0.007,* respectively). The BO procedure caused significant increases in IL-1β, TNF-α, and cytochrome C levels (*p* < *0.05*) in comparison with the control group. BA decreased IL-1β and TNF-α compared to the BO group (*p* < *0.05*). Although it was observed that prophylactic BA application reduced cytochrome c levels, there was no statistically significant difference compared to the BO group.

**Table 4:** Comparisons of proinflammatory cytokines and cytochrome C levels between assay groups

Parameters (mean ± SD)	Group 1 (*n* = 8)	Group 2 (*n* = 8)	Group 3 (*n* = 8)	Group 4 (*n* = 8)	*p*
IL-1β (ng/mL)	10.28 ± 0.05	41.79 ± 1.26^a^	22.83 ± 1.63^ab^	31.02 ± 0.43^abc^	< 0.0001
TNF-α (ng/L)	356.8 ± 16.2	618.1 ± 2.1^a^	419.5 ± 1.3^ab^	510.7 ± 1.4^abc^	< 0.0001
Cytc (ng/L)	759.7 ± 55.2	1028 ± 55.5^a^	963.8 ± 56.5^a^	1005 ± 164.7^a^	0.007

*TNF*-α; Tumor necrosis factor-alpha, *IL-6*; Interleukin-6, *Cytc*; Cytochrome C

Group 1: Control, Group 2: Biliary Obstruction (BO), Group 3: BO + Boric acid prophylaxis, Group 4: BO + Boric acid treatment. Results are expressed as mean ± standard deviation (SD). Since all data were normally distributed, the comparisons of variables among groups were analyzed with the oneway-ANOVA; The P-value shows the differences among all groups. Since all data were passed the homogeneity of variances test, the multiple comparisons between two groups were analyzed with the Bonferroni-post-hoc test; ^a^ = *P* < 0.05 compared with the control group (group 1), ^b^ = *P* < 0.05 compared with the BO group (group 2), ^c^ = *P* < 0.05 compared with the BO + Boric acid prophylaxis group (group 3)

### Liver Tissue TNF-α, Interleukin-6 (IL-6), Caspase-3, and B-cell Lymphoma 2 (Bcl-2) Gene mRNA Expression Levels

**Fig. 1:**
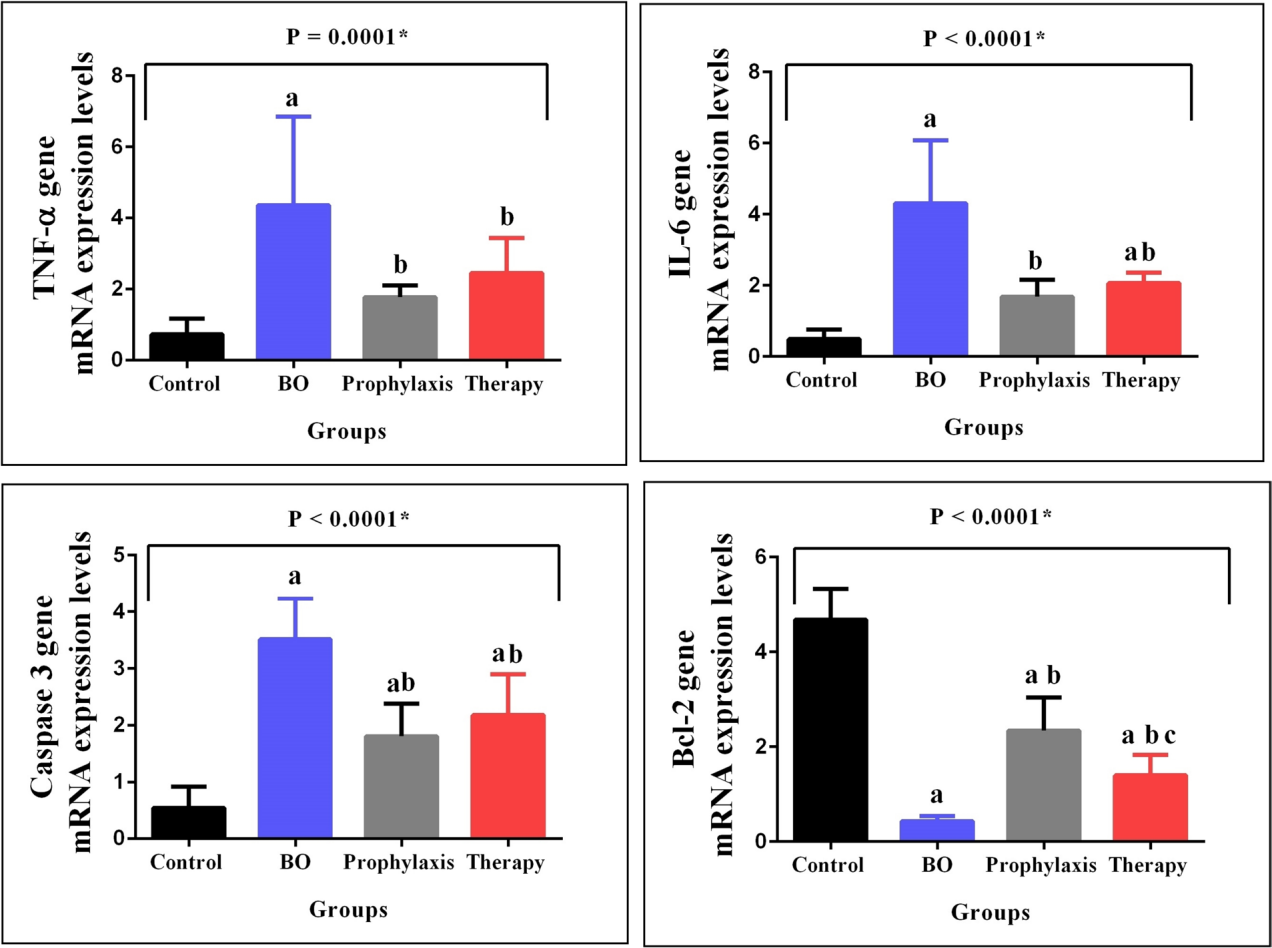
Graphical representation of differences in TNF-α, IL-6, caspase-3 and Bcl-2 gene mRNA expression levels in liver tissue samples of groups. *N* = 8 in each group. Group 1: Control, Group 2: Biliary Obstruction (BO), Group 3: BO + Boric acid prophylaxis, Group 4: BO + Boric acid treatment. Results are expressed as mean ± standard deviation (SD). The P-value shows the differences among all groups (oneway-ANOVA). Statistical significance (Bonferroni-*post-hoc* test) is indicated as follows; a = *P* < 0.05 compared with the control group (group 1), b = *P* < 0.05 compared with the BO group (group 2), c = *P* < 0.05 compared with the BO + Boric acid prophylaxis group (group 3). *Abbreviations: TNF-α: Tumor necrosis factor-alpha, IL-6: Interleukin-6, Bcl-2: B-cell lymphoma 2

Figure 1 display the differences in TNF-α, IL-6, caspase-3, and Bcl-2 gene mRNA expression levels in the liver tissue among assay groups. The mRNA expression levels of TNF-α, IL-6, caspase-3, and Bcl-2 genes showed significant differences among the groups (*p* = *0.0001, p* < *0.0001, p* < *0.0001, p* < *0.0001,* respectively). The BO procedure caused significant increases in TNF-α, IL-6, and caspase-3 gene mRNA expression levels (*p* < *0.05*) and significant decreases in Bcl-2 gene mRNA expression levels (*p* < *0.05*) in comparison with those caused by the control group. BA decreased TNF-α, IL-6, and caspase-3 gene mRNA expression levels and increased Bcl-2 gene mRNA expression levels (Fig. 1).

### Histopathologic Examinations

**Fig. 2:**
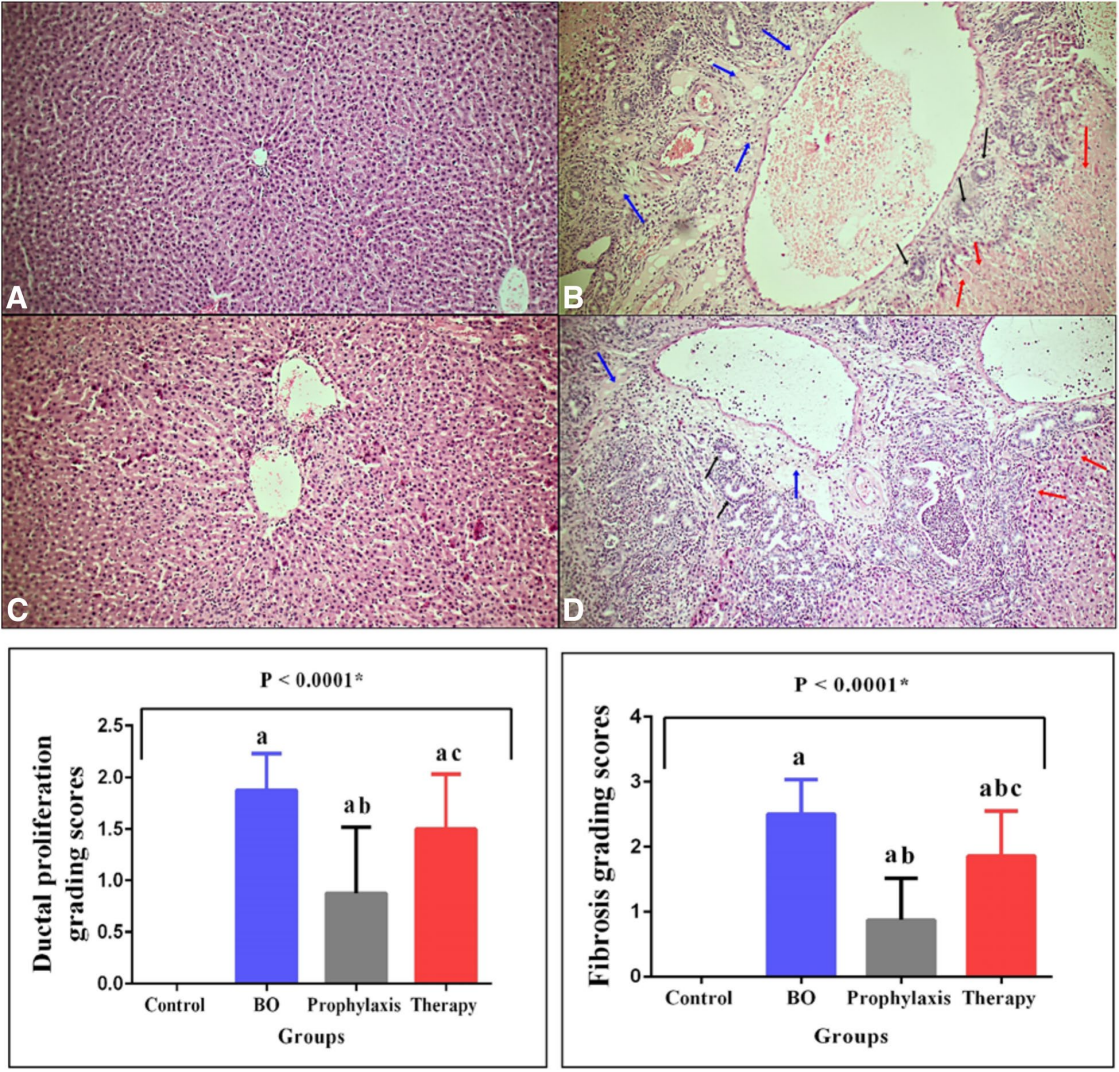
Representative photomicrographs of H&E stained liver tissue samples in groups (H&Ex100). **A**: Control group, **B**: Biliary Obstruction (BO) group, **C**: BO + Boric acid prophylaxis group, **D**: BO + Boric acid treatment group. Black arrow, ductal proliferation; blue arrow, inflammation and fibrosis; red arrow, hepatocyte necrosis. Graphical representation of the differences between the histopathological scoring values ​​of liver samples belonging to the groups. N = 8 in each group. Group 1: Control, Group 2: Biliary Obstruction (BO), Group 3: BO + Boric acid prophylaxis, Group 1: Control, Group 2: Biliary Obstruction (BO), Group 3: BO + Boric acid prophylaxis, Group 4: BO + Boric acid treatment. Results are expressed as mean ± standard deviation (SD). The P-value shows the differences among all groups (oneway-ANOVA). Statistical significance (Bonferroni-*post-hoc* test) is indicated as follows; a = *P* < 0.05 compared with the control group (group 1), b = *P* < 0.05 compared with the BO group (group 2), c = *P* < 0.05 compared with the BO + Boric acid prophylaxis group (group 3)

**Fig. 3:**
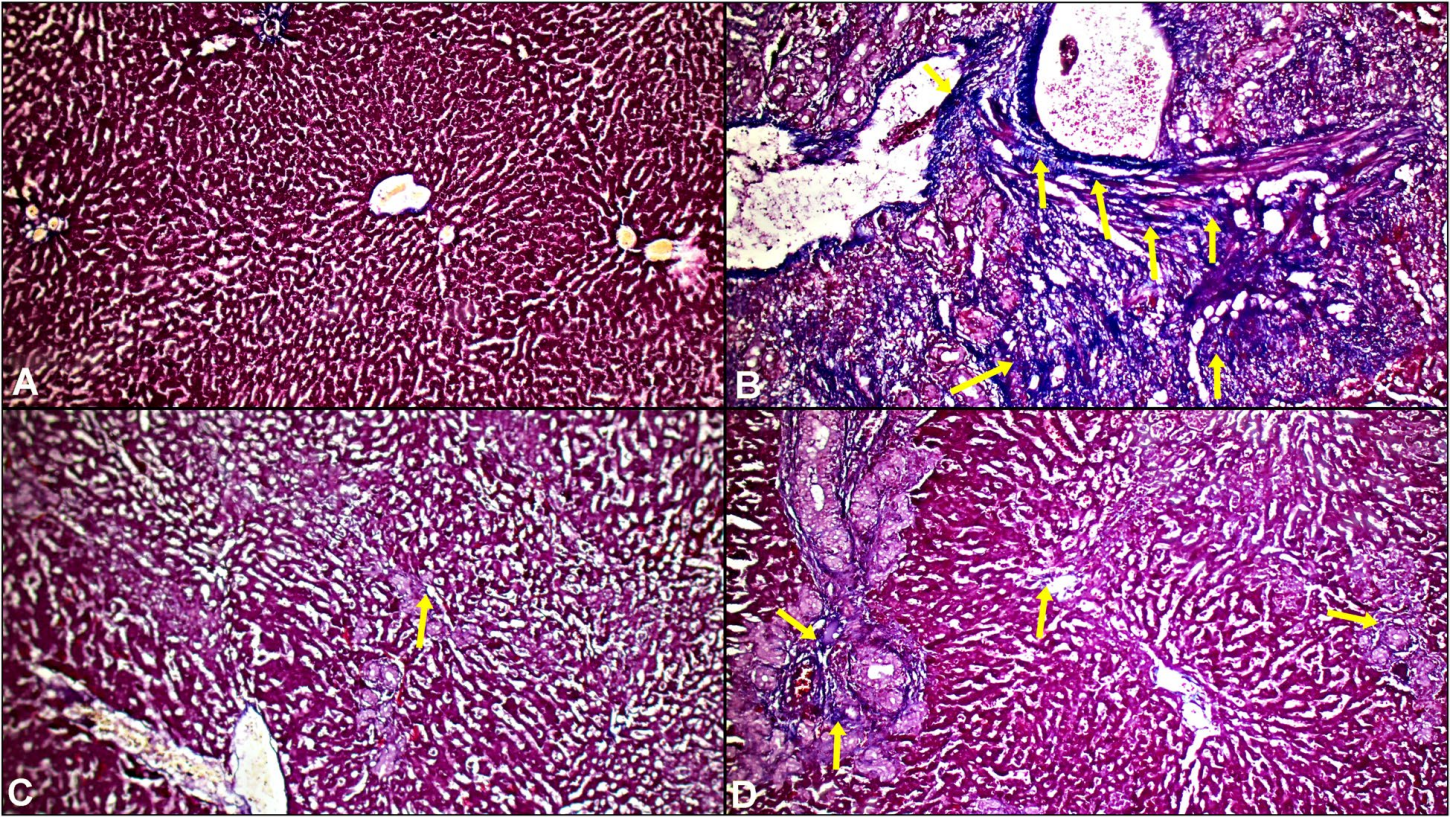
Representative photomicrographs of Masson’s trichrome stained liver tissue samples in groups (Masson’s Trichrome × 100). **A**: Control group, **B**: Biliary Obstruction (BO) group, **C**: BO + Boric acid prophylaxis group, **D**: BO + Boric acid treatment group. Yellow arrow, fibrous and collagenous band areas

Differences in scored histopathological grade values in liver tissue samples among assay groups are represented in Fig. 2. In the BO group, the histopathological changes including, central vein dilatation, fibrosis in the pericentral region, ductal proliferation, and chronic inflammatory cell infiltration, were observed (Fig. 2). Additionally, fibrous and collagen bands with occasional bridging in the liver parenchyma were observed (Fig. 3). Histopathological analysis showed that BA application alleviated the morphological changes caused by the BO procedure, leading to a significant decrease in the histopathological score compared to the control group (*p* < *0.0001*). Although preoperative and postoperative BA application was observed to alleviate the morphological changes caused by the BO procedure, prophylactic BA application was observed to be more effective (Fig. 2).

## Discussion

Disturbance in bile secretion and flow causes cholestasis, which damages the liver by accumulating bilirubin, toxic bile salts, and cholesterol inside the cells. Portal tract dilatation, biliary tract proliferation, hepatic stellate cell activation, extracellular matrix accumulation, liver fibrosis, and related cirrhosis are all signs of bile duct obstruction [22]. Cholestatic liver injury is one of the most important causes of fibrotic liver. Increased liver and serum bile acid levels lead to liver toxicity and subsequently fibrosis and cirrhosis. Cholestatic liver fibrosis is believed to be initiated and progressed in part by activation of biliary proliferation [23].

In this study, we assessed the prophylactic and therapeutic effects of BA in a biliary obstructive cholestasis rat model. According to our findings, BA considerably prevents BO-induced liver damage and fibrosis, as shown by the decrease in high serum AST and ALT activities as well as the reduction in bile duct proliferation, liver inflammation, and fibrosis. BA reduced the increase in serum enzyme activities and bilirubin levels. By histopathological examination of liver tissue samples, it was observed that BA significantly reduced inflammation, ductal proliferation, and fibrosis. BA application showed anti-inflammatory effect by reducing proinflammatory cytokine levels, anti-oxidative effect by reducing oxidative stress parameters, and anti-apoptotic effect by reducing apoptotic gene expression. Although BA is effective both prophylactically and therapeutically, it was observed that its prophylactic effect was more effective.

Mitochondria are the main organelles in the harmful effects of bile acids, because bile acids directly cause mitochondrial dysfunction [24]. Research has demonstrated that cholestatic liver damage and its associated oxidative stress, inflammation, and cell death are largely caused by mitochondrial malfunction [4, 25, 26]. By increasing mitochondrial permeability and releasing cytochrome c, mitochondria contribute significantly to both necrotic and apoptotic cell death. It has been demonstrated that cytochrome c accumulation in the cytoplasm triggers the activation of caspase and DNA breakage enzymes, which cause apoptotic cell death [27]. Miller et al. reported that cytochrome c is a useful indicator of liver injury, supporting that it may be useful as non invasive biomarkers of hepatotoxicity [28]. In our study, it was observed that the BO procedure caused a significant increase in cytochrome C levels.

Measurement of liver enzyme activities secreted into the blood by the damaged hepatocyte is the most useful tool available for detecting liver damage. Liver panel tests such as bilirubin, ALP, AST, ALT, and GGT are routinely used to detect liver damage [29]. In this study, we observed that mechanical cholestasis caused a severe increase in bilirubin levels and AST, ALT, ALP, GGT, and amylase activities. BA caused a significant decrease in bilirubin levels and these enzyme activities. Especially in the prophylactic group, where BA was applied before the BO procedure, the improvement in enzyme activities and bilirubin levels was more remarkable compared to the therapeutic group. In a recent study examining the prophylactic and therapeutic efficacy of BA in lipopolysaccharide-induced liver inflammation in rats, prophylactic BA administration caused a significant decrease in AST, ALT, ALP, and GGT activities [30]. Similar to our findings, in a study examining the protective effect of BA against aluminum-induced hepatotoxicity in rats, BA pretreatment was shown to cause a decrease in AST, ALT, and ALP activities [31].

Oxidative stress has been associated with the development of cholestatic liver injury and is a key feature of obstructive cholestasis [32]. Oxidative stress has been shown to increase in rats with cholestasis 24 h after bile duct ligation [33]. It has been suggested that BA exhibits antioxidant effects against oxidant compounds by quenching and breaking the chains through the protons in the hydroxyl groups [16]. In our study, we examined the antioxidant effect of BA by measuring TAS, which shows the total effect of all antioxidants, TOS, which shows the total effect of oxidants, and by calculating OSI levels, which is a marker of oxidative stress. We found that BA application decreased TOS and OSI levels and increased TAS levels. Similarly our results, in mice with CCl(4)-induced liver damage, Ince et al. found that BA administration markedly raised the glutathione content as well as the activities of catalase and superoxide dismutase in the liver. They suggested that boric acid has strong hepatoprotective effects by increasing the activity of the antioxidant defense system [34]. A recent study has shown that BA has antioxidant effects in the rat liver tissue by reducing malondialdehyde levels and increasing superoxide dismutase and catalase enzyme activities [35]. In line with our findings, BA pretreatment enhanced antioxidant capacity and lowered oxidant capacity in a study investigating the protective effects of BA in a rat model of experimental cholestatic liver ischemia reperfusion injury [36]. In a study, BA was shown to increase liver tissue malondialdehyde levels and superoxide dismutase, glutathione peroxidase, and catalase activities in cyclophosphamide-induced liver damage in rats [37]. Pawa et al. demonstrated that boron in the form of borax pretreatment normalized the liver by regulating oxidative stress parameters in experimental fulminant liver failure induced by thioacetamide in rats [38]. The affinity of BA for hydroxyl groups may be the mechanism explaining the biological effects of BA, especially antioxidant [39].

Pro-inflammatory cytokines are strong inducers of intrahepatic cholestasis, especially TNF-α and IL-2 [40]. In our study, we measured serum TNF-α, IL-1β levels and liver tissue IL-6, TNF-α mRNA gene expression levels to examine the anti-inflammatory effect of BA. While biliary obstructive cholestasis caused a significant increase in proinflammatory cytokine levels, BA administration was observed to cause a decrease in serum TNF-α, IL-1β and liver tissue TNF-α, IL-6 gene expression levels. The anti-inflammatory effect of BA was more pronounced in the prophylactic group. Consistent with the results of this study, in a study investigating the protective effects of boron against the toxic effects of cyclophosphamide on the liver in rats, a significant decrease in serum NF-kB, TNF-α, IL-1β, IL-6 levels and an increase in IL-10 levels were found [41]. Basbug et al. reported that BA administered prophylactically before the procedure had a protective effect by reducing tumor TNF-α and IL-6 levels in a liver injury model caused by hepatic ischemia–reperfusion in rats [42]. The results of our study revealed that BA may have an anti-inflammatory effect by suppressing proinflammatory mediators, in accordance with the literature [16, 30, 43, 44].

Apoptosis brought on by bile acids is a significant factor in cholestatic liver disease. Bile acids and other molecules such as bilirubin, known as mitochondrial toxins that accumulate in the body during cholestasis, can damage the mitochondria [45, 46]. Cholestasis-induced organ damage has been linked to increased mitochondrial membrane permeability, mitochondria-facilitated reactive oxygen species production, abnormalities in mitochondrial energy metabolism, and mitochondria-mediated cell death [47, 48]. The induction of apoptosis by hydrophobic bile acids is thought to be due to an imbalance between proapoptotic and antiapoptotic signaling pathways [49]. In various studies, toxic bile acids have been shown to induce hepatocyte apoptosis [50, 51]. In this study, it was shown that biliary obstructive cholestasis increased caspase-3 mRNA gene expression levels and decreased Bcl-2 mRNA gene expression levels. While BA application decreased caspase-3 gene expression levels, it caused an increase in Bcl-2 gene expression levels. Kurosawa et al. showed that during extrahepatic cholestasis in rats, the potent antiapoptotic protein Bcl-2 was expressed hepatocellularly [52]. Miyoshi et al. assessed apoptosis in cholestatic rat liver using caspase-3 immunostaining and activity, and they found that apoptosis was markedly elevated in the first week following bile duct ligation. They reported that hepatocyte apoptosis during cholestasis plays a role in liver injury [53]. Another study showed that the ratio of BAX and BCL-2 increased 3 days after bile duct ligation during cholestasis in rats [54]. In our study, while BA administration decreased caspase-3 gene expression levels, it caused an increase in Bcl-2 gene expression levels. It was demonstrated that BA administration raised the expression levels of the Bcl-2 protein while decreasing those of the Bax and caspase-3 proteins in a study investigating the protective effect of BA on acrylamide-induced acute liver injury in rats [55]. Our study’s findings, which are in line with previous research, demonstrated that BA effectively prevented cholestasis-induced apoptosis by increasing anti-apoptotic proteins and decreasing apoptotic proteins [41, 42, 56, 57].

Histopathological examination results showed that cholestasis due to experimental biliary obstruction caused ductal proliferation, severe fibrosis, congestion, neutrophilic cell infiltration, and hepatocyte necrosis in the liver tissue. Additionally, loss of intercellular border loss in hepatocytes, nuclear pycnosis, and cytoplasmic eosinophilia were observed in the biliary obstruction group. BA significantly reduced inflammation, ductal proliferation and fibrosis. The improvement in liver tissue was more evident in the prophylaxis group than in the treatment group. The histopathological findings of our study revealed that BA ameliorated cholestasis-induced liver damage, consistent with previous studies [8, 30, 31, 34, 36–38, 41–43, 55–57]. Limitations of the current study include the possible effect of dose differences due to the use of only a single dose of BA. Additionally, the current study examined short-term hepatic damage caused by cholestasis without evaluating long-term outcomes.

## Conclusion

To our knowledge, this is the first experimental study in the literature examining the effect of BA on the mechanical biliary obstructive cholestasis rat model. The histopathological, biochemical, and molecular analysis results of this study showed that BA exhibited hepatoprotective effects in rats in which we experimentally developed a biliary obstructive cholestatic liver model. BA reduced the release of inflammatory mediators, OSI levels, and apoptotic caspase 3 gene mRNA expressions. In addition, BA increased antiapoptotic Bcl-2 gene mRNA expressions. These effects were accompanied by improvements in bilirubin levels, hepatobiliary enzyme activities, and liver histopathology, such as hepatocyte necrosis, ductal proliferation, and fibrosis. It has been observed that the prophylactic application of BA before the procedure is more effective than the therapeutic application after the procedure. More comprehensive experimental molecular and clinical studies are needed to better understand the mechanisms behind this preventive effect of BA. It is thought that it would be useful to investigate BA administration with pharmacokinetic studies examining different dose applications and long-term effects, especially in cholestasis-related liver damage.

## Data Availability

No datasets were generated or analysed during the current study.
